# Structure, Application, and Biochemistry of Microbial Keratinases

**DOI:** 10.3389/fmicb.2021.674345

**Published:** 2021-06-23

**Authors:** Qingxin Li

**Affiliations:** Guangdong Provincial Engineering Laboratory of Biomass High Value Utilization, Institute of Bioengineering, Guangdong Academy of Sciences, Guangzhou, China

**Keywords:** keratinase, waste treatment, keratin, microorganisms, structure, protease

## Abstract

Keratinases belong to a class of proteases that are able to degrade keratins into amino acids. Microbial keratinases play important roles in turning keratin-containing wastes into value-added products by participating in the degradation of keratin. Keratin is found in human and animal hard tissues, and its complicated structures make it resistant to degradation by common proteases. Although breaking disulfide bonds are involved in keratin degradation, keratinase is responsible for the cleavage of peptides, making it attractive in pharmaceutical and feather industries. Keratinase can serve as an important tool to convert keratin-rich wastes such as feathers from poultry industry into diverse products applicable to many fields. Despite of some progress made in isolating keratinase-producing microorganisms, structural studies of keratinases, and biochemical characterization of these enzymes, effort is still required to expand the biotechnological application of keratinase in diverse fields by identifying more keratinases, understanding the mechanism of action and constructing more active enzymes through molecular biology and protein engineering. Herein, this review covers structures, applications, biochemistry of microbial keratinases, and strategies to improve its efficiency in keratin degradation.

## Introduction

Keratin is an important structural protein in some hard tissues in which it plays a protective role by forming a barrier between the organ and its environment. Keratin is a fibrous protein that is insoluble in water and other solvents. Due to the structure of keratin stabilized by disulfide bonds and hydrogen bonds, keratin is resistant to degradation by common proteases such as trypsin and pepsin. Keratin is one of the ubiquitous proteins in nature and found in many organs such as feather of birds, hair, wools, and nails of mammals (McKittrick et al., 2012; Chilakamarry et al., 2021). Keratin is among the most abundant renewable organic polymers in nature after cellulose, lignin, hemicellulose, pectin, and chitin (Lange et al., 2016; Bealer et al., 2020). Keratin-containing wastes such as feathers from poultry industry represent an attractive resource for carbon, sulfur, and nitrogen that can be converted into other products (Bhari et al., 2021).

Keratinous wastes are rich in amino acids (Qiu et al., 2020) and could affect the atmosphere, water sources, and soil if they are not treated properly (Hassan et al., 2020). On the other hand, this type of wastes serves as a low-cost resource for amino acids or can be converted into animal feeds and fertilizers (Pettett and Ipek, 2004; Gurav and Jadhav, 2013). Compared with other natural polymers such as cellulose, starch, and collagen, extraction of keratin is a challenging process. Quite a few strategies such as physical, chemical, and biological methods are applied in keratin extraction. Although chemical and physical treatments are efficient strategies to treat keratinous wastes, a large amount of energy is needed and amino acids were destroyed during treatment. As keratin does not accumulate in nature, microorganisms are playing the major role in its degradation and recycling. Therefore, keratinous wastes threatening the environment can be converted into value-added products by using microbial treatment (de Menezes et al., 2021; Nnolim and Nwodo, 2021). Extensive studies have been carried out to search suitable microorganisms and obtain optimized processes to make full use of keratinous wastes (Gradišar et al., 2000; Sangali and Brandelli, 2000; Kim et al., 2001; Rai and Mukherjee, 2011). It has been shown that wastes such as feathers can be degraded by bacteria and fungi to produce other important products such as amino acids or proteins with added values (Callegaro et al., 2018; Shanmugasundaram et al., 2018; Bohacz, 2019; Tamreihao et al., 2019; Chaudhary et al., 2021). Therefore, conversion of the wastes using microorganisms is the most environmentally friendly method while more studies are still needed to improve the degradation efficiency of keratins. As the amount of keratin-containing wastes is increasing rapidly due to various reasons, keratin derived from the wastes should be fully utilized by serving as a source of proteins, amino acids, and a low-cost resource for producing other products.

The structure of keratin explains their relatively stable existence and resistance to chemicals. Keratin can be classified as α-keratin and β-keratin according to the composition of amino acids and the secondary structure of polypeptide chains (Fraser and Parry, 2008, 2011; Lange et al., 2016). It is shown that α-keratin is mainly present in mammals and β-keratin is in avian and reptilian tissues. The polypeptide chains are packed into the final structure through disulfide bonds formed by cysteine residues, hydrogen bonds, and hydrophobic interactions (Vidmar and Vodovnik, 2018). Cysteine residues play a key role in the structural stability of keratin by forming intra- or intermolecular disulfide bonds (Barone et al., 2005). Keratin is also classified as hard keratin and soft keratin based on the content of cysteine (Jin et al., 2017). As basic units of keratin are polypeptides, keratinases play a major role in keratin degradation by breaking the disulfide bonds and peptidic bonds (Fraser and Parry, 2008; Hassan et al., 2020; de Menezes et al., 2021). One of the features of keratinases is that they are able to cleave a sequence with hydrophobic residues at the P1 position (Brandelli et al., 2010). Most keratinases were reported to degrade keratins in the presence of disulfide reducers or reducing agents while such reducing environment should not be required for a true keratinase (Qiu et al., 2020). Currently, the identified keratinases produced by microorganisms can be classified into at least 14 protease families (Qiu et al., 2020). In addition to journal publications, quite a few patent literatures reported preparation, extraction, and recombinant production of keratinases. The related patents can be obtained from different resources (Yahaya et al., 2021). Over 20,000 records can be obtained in google patent when keratinase was used as a searching keyword^1^. In this review, the structure and function of keratinases from bacteria and fungi are discussed. With accumulated knowledge in understanding microbial degradation of keratin, keratin-rich wastes are considered as a valuable and low-cost resource that can be converted into diverse products such as feed and fertilizers.

## Mechanism of Action for Keratinase

Keratinases can be understood as a class of proteases that are able to degrade keratin by cleaving the peptide bonds (Gupta and Ramnani, 2006; Sahni et al., 2015). Although the identified keratinases are serine and metalloproteases that are able to break the peptide bond in peptide chains, they recognize hydrophobic substrates and affect the disulfide bonds. Most keratinases require other enzymes to break the disulfide, and two steps, namely, keratin peptide releasing and peptide degradation are included in keratin degradation. Reduction reaction can be catalyzed by disulfide reductases or reducing agents (Sangali and Brandelli, 2000; Rahayu et al., 2012; Lange et al., 2016). Microbial keratinases are usually secreted into the medium when the microorganism was cultivated in a keratin-containing medium (Friedrich et al., 1999; Monod, 2008; Jayalakshmi et al., 2010). This is not surprising as keratins are not soluble and not able to be transported into cells. Studies have shown that diverse keratinases with different molecular weights, optimal pH values, and optimal temperatures are produced by microorganisms. Most microbial keratinases are secreted into the extracellular matrix in the presence of keratin or keratin-containing substrates (Vidmar and Vodovnik, 2018; Nnolim and Nwodo, 2021). Some microorganisms are able to produce extracellular and intracellular keratinases simultaneously. Cell-bound keratinases are also identified, and this type of enzymes might be of great interest for industrial application as well, which is due to the fact that they are immobilized on the cell surface and can be easily used in waste treatment. With the development of molecular biology and accumulation of genome sequences of microorganisms, microbial keratinases and their mechanism of keratin degradation can be predicted through bioinformatics (Li et al., 2018; Song et al., 2018; Pinski et al., 2020; Zolfaghari Emameh et al., 2021). A recent report showing a careful and detail classification of current keratinases provides a clear view to understand their mechanism of action and explains the requirement of multiple enzymes to achieve complete degradation of keratin or keratinous wastes (Qiu et al., 2020).

### Keratinase-Producing Microorganisms

**FIGURE 1:**
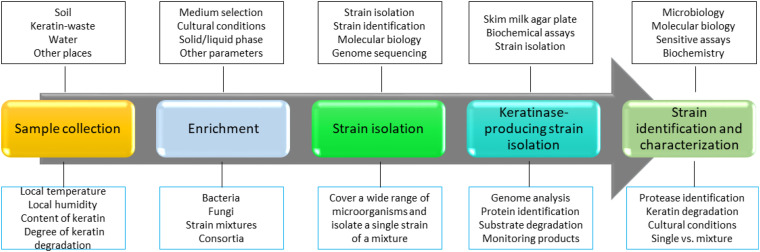
A strategy to screen keratinase-producing microorganisms. In this strategy, a method to identify keratinase-producing strain is critical. Molecular biology, bioinformatics, biochemistry, and sensitive analytical methods such as MS are critical in the screening. In addition, as keratin degradation is a complicated step, sample isolation and enriching steps are important to make sure that the desired strains are sustained. Many keratinase-producing strains have been isolated and identified from the environment (Nnolim and Nwodo, 2021).

Keratinase-producing microorganisms are widely distributed in nature, and they can be readily isolated from the environment (Vidmar and Vodovnik, 2018). Bacteria, fungi, and actinobacteria are able to produce keratinases and use keratin as the carbon and nitrogen sources in the minimal medium. To isolate a keratinase-producing microorganism, keratins or keratin-containing wastes such as feathers are usually present in the cultural medium, implying that microbial keratinase production is an inducible process (Brandelli et al., 2010). To isolate keratinase-producing microorganisms, the following steps including sample collection, assay development, strain identification, and characterization are usually applied (Figure 1). First, samples need to be collected from the environment. The samples can be soils, keratin-containing wastes, or water that was contaminated with wastes (Jeevana Lakshmi et al., 2013; Gegeckas et al., 2014). Feathers are one of the most commonly used substrates in screening, and degradation of feathers can be readily monitored by observing the changes in shapes and releasing of proteins into the solution. It has been noted that several factors such as location, water content, keratin composition, and weather of the local environment need to be considered to make sure that a collection of microorganisms can be obtained. Second, a medium for the microorganism growth needs to be set up. As the media for enriching bacteria or fungi are different, a suitable medium is important in the screening (De Azeredo et al., 2006; Fakhfakh et al., 2011; Mazotto et al., 2013; Parrado et al., 2014; Arokiyaraj et al., 2019). In addition, other parameters such as cultural time and temperature should be considered based on the experimental objectives. For example, if a team plans to isolate a thermally stable keratinase, a higher temperature in screening might enhance the rate of success (Wu et al., 2017). In the case of isolating microbial consortia, the enrichment step might not be important as it might reduce the content of certain microorganisms. Third, an assay for measuring protease activity should be set up for ranking keratin degradation efficiencies caused by different keratinases (Iglesias et al., 2017). It will be useful to monitor both changes in the shape of keratin substrate and the composition of the proteins secreted by the microorganisms. Last, a reliable method for microorganism identification is needed (Herzog et al., 2016). In addition to identifying the amino acid sequence of the enzyme through analyzing the MS data carefully, knowing the genome of the screened microorganisms will provide more information to understand mechanism of action for the identified keratinase and a strategy to express certain types of enzymes (Sittipol et al., 2021).

*Bacillus* stains are the predominate bacteria that are able to produce keratinases. The species include *Bacillus subtilis*, *Bacillus pumilus*, *Bacillus lichenifomis*, and *Bacillus cereus* (Zaraî Jaouadi et al., 2015; Bhari et al., 2018; Gegeckas et al., 2018). Some other bacteria are able to degrade feathers with a high efficiency (Kim et al., 2005; Zaraî Jaouadi et al., 2015; Abdel-Naby et al., 2017; Arokiyaraj et al., 2019; Hamiche et al., 2019). Keratin-degrading fungi can be isolated from some tissues of human and animals as the produced keratinase might be important for the fungal infection (Friedrich et al., 1999; Bohacz and Korniłłowicz-Kowalska, 2019; Zhang et al., 2019). It was shown that fungi such as *Chrysosporium* (Bohacz, 2016; Gurung et al., 2018) and *Trichophyton* (Zhang et al., 2019) were able to degrade keratins through their produced keratinases (Shadzi et al., 2002; Hassan et al., 2020). Due to the function of keratins, keratinases in some pathogenic fungi might be essential for the invasion by breaking the barrier between the tissue and the environment. *Streptomyces* is the predominant actinobacterium that is able to produce keratinases (Li et al., 2020). Quite a few reports have shown that keratinolytic actinobacteria can be isolated from different environment. Some actinobacteria are able to produce thermally stable keratinases which have great potential to be widely used in industry (Nnolim and Nwodo, 2021). The detail introduction of keratinase-producing microorganisms have been described in several reviews (Papadopoulos, 1989; Onifade et al., 1998; Korniłłowicz-Kowalska and Bohacz, 2011; McKittrick et al., 2012; Sahni et al., 2015; Kanoksilapatham and Intagun, 2017; Pahua-Ramos et al., 2017; Verma et al., 2017; Kowalczyk et al., 2018; Li et al., 2018; Vidmar and Vodovnik, 2018; Li, 2019; Hassan et al., 2020; Kumar, 2020; Qiu et al., 2020; Yahaya et al., 2021).

Although many keratinase producers have been isolated and identified (Cavello et al., 2020; Jagadeesan et al., 2020; Moridshahi et al., 2020; Nnolim et al., 2020b; Reis et al., 2020), the isolation and characterization of keratinase-producing microorganisms are still an important task. The keratin degradation efficiency can be improved when more keratinases are applied (Peng et al., 2019). Therefore, a mixture of microorganism-microbial consortia might have great potential in converting keratin-rich waste into valuable products (Kang et al., 2020; Nasipuri et al., 2020). It is challenging to have a microbial consortium because the amount of the organism in the system will be affected under different conditions. It is also possible to set up a microbial consortium to improve keratin degradation by mixing several microorganisms which have been well characterized. This is a feasible method in industrial applications.

### Keratin Degradation by Keratinases

**FIGURE 2:**
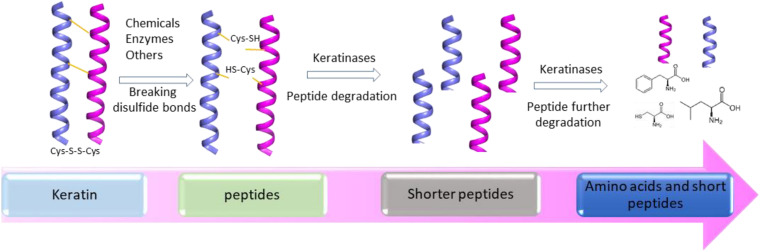
A simplified diagram showing the degradation of keratin by proteases. Two-step disulfide bond breakage and polypeptide degradation are usually included in keratin degradation. Keratins are simplified as helices. The degradation includes the steps such as releasing of keratin and degradation of keratin by multiple enzymes, which has been described in several reviews (Korniłłowicz-Kowalska and Bohacz, 2011; Sharma and Devi, 2018; Vidmar and Vodovnik, 2018; Qiu et al., 2020).

Several mechanisms have been proposed based on the accumulated studies (Korniłłowicz-Kowalska and Bohacz, 2011; Li et al., 2020). The challenge in keratin degradation is due to the presence of high content of disulfide bonds (Korniłłowicz-Kowalska and Bohacz, 2011). As most keratinases are proteases responsible for breaking peptide bonds, other enzymes or chemicals are needed to affect the disulfide bonds and reduce the forces for keratin packing to make proteins accessible to the proteases (Kasperova et al., 2013). Therefore, at least two steps including disulfide bond breakage and proteolysis are involved in keratin degradation (Figure 2). It has been noted that fungi and bacteria use similar ways to degrade keratins with a slight difference as mechanical destruction is also playing a role in degradation by fungi (Korniłłowicz-Kowalska and Bohacz, 2011). It was shown that mechanical destruction, production of inorganic sulfite, and involvement of disulfide reductase can contribute to the step of disulfide bond breakage (Korniłłowicz-Kowalska and Bohacz, 2011). In the protein degradation step, keratinases are able to break polypeptides into amino acids. There are several protease families responsible for keratin degradation (Qiu et al., 2020). To convert keratin into amino acids completely, multiple keratinases are required as different enzymes prefer to different cleavage sites. The diversity and classification of keratinase based on the amino acid sequence and conserved domains in MEROPS have been described in detail in a recent review (Qiu et al., 2020). Some disulfide reductases involved in keratin degradation were shown (Kasperova et al., 2013; Navone and Speight, 2018; Table 1). The roles of these enzymes in keratin degradation have been described in other literatures, which will not be described here (Kasperova et al., 2013; Lange et al., 2016; Mercer and Stewart, 2018; Flückger et al., 2020).

**TABLE 1:** Some studies on other enzymes important for keratin degradation are listed.

Organism	Remarks	References
*Bacillus halodurans* PPKS-2	This strain produces multiple enzymes such as keratinases and disulfide reductase.	Prakash et al., 2010b
*Stenotrophomonas* sp. D-1	Cooperative action of a protease and a reductase was observed.	Yamamura et al., 2002
*Arthroderma benhamiae*	Sulfite can be produced from cysteine by cysteine dioxygenase Cdo1.	Grumbt et al., 2013
*Bacillus licheniformis* RG1	Multiple enzymes are shown to be important for keratin degradation.	Ramnani et al., 2005
*Trichophyton mentagrophytes*	Cysteine dioxygenase expression was studied.	Kasperova et al., 2013, 2014
*Trichophyton mentagrophytes*	A gene for expressing cysteine dioxygenase was obtained.	Kasperova et al., 2011
*Bacillus* sp. MTS	Cysteine reductase improved keratin degradation.	Rahayu et al., 2012
Keratin-degrading fungi	A study showed the classification of lytic polysaccharide monooxygenases.	Busk and Lange, 2015
*Streptomyces pactum*	Keratinases and reduction of disulfide bonds are important for feather degradation.	Bockle and Muller, 1997

Accumulated studies have shown that the crude microbial culture exhibited higher keratin degradation efficiency than the purified enzymes. A keratinase-degrading system can be developed by carefully analyzing the components or enzymes that are critical for keratin degradation. The crude culture of a microorganism is a mixture of enzymes which can be used in keratin treatment. Therefore, a mixture of enzymes can be readily obtained by exploring the effects of cultural conditions on keratin degradation. Two important elements are important in this strategy. One is to have a good strain to work with and the other is to have an optimized fermentation condition to produce an enzymatic system for keratin degradation.

### Structure of Keratinase

Keratinases are serine and metalloproteases, and their active sites are formed by several conserved residues. Crystal structures of several keratinases demonstrate the structural basis for their activity and provide insights into designing more stable and efficient enzymes for industrial applications (Betzel et al., 2001; Gupta et al., 2017). These structures can also be utilized as a template to obtain homology models of other keratinases (Kluskens et al., 2002).

**FIGURE 3:**
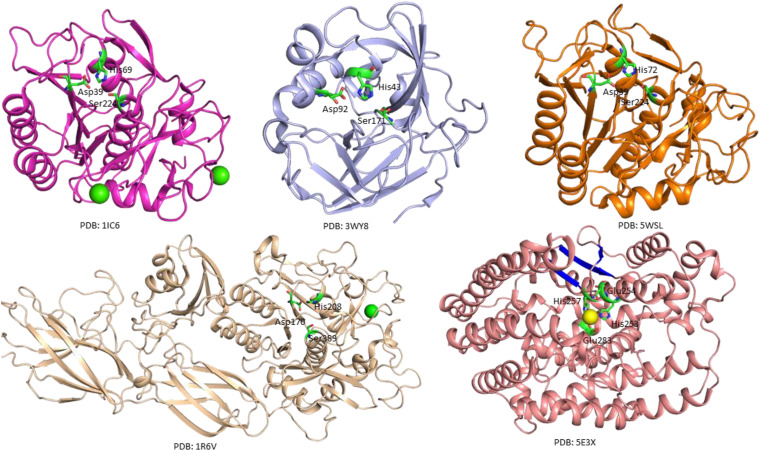
Crystal structures of microbial keratinases. The crystal structures of five keratinases were shown to understand their mechanism of action. The PDB access codes of the structures are indicated. The amino acids in active sites are highlighted in sticks and labeled with sequence numbers. Ca^2+^ and Co^2+^ atoms in the structures are shown as yellow and green spheres, respectively. In the crystal structure of fervidolyis (PDB:1R6V) (Kim et al., 2004), His208 was mutated into Ala. Ala 208 was labeled as His208 in this figure to show the active site. The β-sheet structures in FisCP (PDB: 5E3X) are highlighted in blue (Lee et al., 2015). All the figures were made using PyMOL (www.pymol.org). The details of the structures can be found in the reports (Betzel et al., 2001; Kim et al., 2004; Teruo et al., 2015; Wu et al., 2017).

**FIGURE 4:**
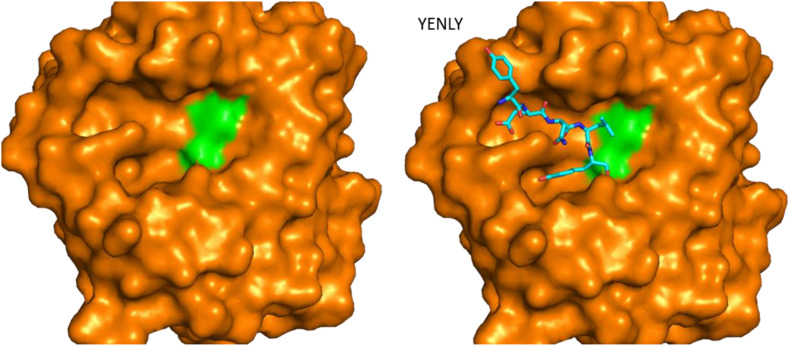
Structure of rMtaKer and its insights into protease and substrate interactions. Surface presentation of one keratinase in the absence and presence of a peptide sequence binding to the active site. The crystal structure of the protease (PDB ID 5WSL) is shown (Wu et al., 2017). The orientation of the figure is similar to those in Figure 3. The residues forming the catalytic triad are shown in green, and the peptide from the adjacent molecule in the crystal structure is shown in sticks. The peptide sequence is shown as sticks in the figure.

The crystal structure of a serine protease from family S8 produced by *Tritirachium album* was obtained (Figure 3; Betzel et al., 2001). Crystal structures of other two members, namely, Fervidolysin from *Fervidobacterium pennivorans* (Kim et al., 2004) and rMtaKer from *Meiothermus taiwanensis* WR-220 (Wu et al., 2017) in S8 family were also determined (Figure 3). Proteases in this family contain both α and β structures. Despite their difference in overall folding, these proteases contain a conserved catalytic triad formed by Asp, His, and Ser residues which are critical for the cleavage of peptide bonds (Figure 3). In addition to determining the folding of the active site in this type of proteases, the structure of rMtaKer provides the molecular basis for substrate and protease interactions (Wu et al., 2017). The molecular interaction between P1–P4 residues and the protease was well resolved, which paved a way to design a suitable substrate for enzymatic assays (Wu et al., 2017; Figure 4). In addition, the structures of these proteases also provide insights into the structural basis for the thermal stability of the enzyme, which is useful for improving the stability of keratinases through site-directed mutagenesis.

A crystal structure of keratin-degrading enzyme from *Paenarthrobacter nicotinovorans* was determined (Figure 3; Teruo et al., 2015). This protease consists of mainly β-sheets to form two β-barrels. The active site with the catalytic triad formed by His43, Asp92, and Ser171 is located between these two barrels (Teruo et al., 2015). The catalytic mechanism of these serine proteases has been well described. The following steps are critical for substrate degradation. The substrate needs to bind to the active site, followed by the peptide cleavage and release of the shorter segments. Proteases from M32 family have different secondary structures. A crystal structure of FisCP from *Fervidobacterium islandicum* AW-1 showed that the protease contained main helical structures with a short β-sheet close to the active site. The active site contains several amino acids (His253, Glue254, His257, and Glue283) and a Co^2+^ atom which is important for coordination of the substrate (Figure 3).

### Biochemistry of Keratinases

Based on the identified enzymes and the sequences deposited in databases, the molecular weights of keratinase range from 20 to 130 kDa (Kanoksilapatham and Intagun, 2017; Sharma and Devi, 2018; Pinski et al., 2020; Qiu et al., 2020). The optimal conditions for enzymatic activity are diverse as different enzymes prefer different pH values, temperatures, and substrates (Tatineni et al., 2007; Tiwary and Gupta, 2010b; Rai and Mukherjee, 2011; Khodayari and Kafilzadeh, 2018; Arokiyaraj et al., 2019; Nnolim et al., 2020a). The classification of keratinases based on the amino acid sequence provides a unique and clear view toward the function and mechanism of keratinases (Qiu et al., 2020), which suggests that obtaining the amino acid sequence of the newly identified keratinases is an important task. Although the effect of keratinase producers on breaking disulfide bonds should be measured (Jaouadi et al., 2010), most studies explored the capabilities of breaking peptide bonds by keratinases (Benkiar et al., 2013; Jaouadi et al., 2013; Ben Elhoul et al., 2016; Omrane Benmrad et al., 2019). To validate the capability to degrade keratin, several substrates were adopted in the enzymatic assay (Böckle et al., 1995; Brandelli et al., 2010; Table 2).

**TABLE 2:** Substrates used for analyzing keratinase activity.

Substrate	Assay	References
Azo-keratin	Measuring absorbance at 450 nm	Herzog et al., 2016; Gonzalo et al., 2020
Keratin azure		Tork et al., 2013, 2016
Feather	Measuring absorbance at 280 nm	Bohacz and Korniłłowicz-Kowalska, 2019
Feather powder		Fang et al., 2013; Qu et al., 2018
Cow horn		Dozie et al., 1994
Wool top		Iglesias et al., 2017
Recombinant feather keratin		Jin et al., 2017
Human hair	Measuring absorbance at 280 nm or using other reagents	Lowry et al., 1951; Gurung et al., 2018
Azocasein	Measuring absorbance at 366 nm	Tork et al., 2013, 2016
Suc-Ala-Ala-Pro-Phe-pNA	Mearing absorbance at 405 nm	Böckle et al., 1995; Mitsuiki et al., 2004; Fang et al., 2016a
Suc-Ala-Ala-Pro-Leu-pNA		Fang et al., 2016b
Bz-Arg-pNA		Böckle et al., 1995
Bz-Phe-Val-Arg-pNa		Rozs et al., 2001
Bz-Ile-Gly-Glu-Arg-pNA		Macedo et al., 2008
Suc-Leu-Leu-Val-Try-AMC (tetrapeptide)	Fluorescence assay	Bakhtiar et al., 2005
Leu-AMC		Monod et al., 2005; Silveira et al., 2009; Brandelli et al., 2010
Short peptides	Reversed-phase chromatography	Mäkinen et al., 1994
Casein	Absorbance at 660 nm	Demirkan et al., 2020

Two types of the substrate were frequently used in the assays (Table 2). One type is the natural keratin such as feathers, wool, and pig bristles or the substrate derived from keratin-rich materials (Laba and Rodziewicz, 2014; Jin et al., 2017). As the solubility of the natural keratin is low, it is challenging to compare the enzymatic activities of keratinases obtained from different studies. Azo-keratin is frequently used as a substrate of keratinase and can be prepared using keratin derived from different sources (Herzog et al., 2016; Gonzalo et al., 2020). Azo-keratin is not a commercial product and is prepared by coupling keratins with a diazotized aryl amide to result in a deep red-orange compound (Riffel et al., 2003). The activity of keratinase is obtained by measuring changes of absorbance at 450 nm. Azo-keratin derived from different keratin-containing materials will be different and might not be an ideal substrate to compare enzymatic activities obtained from different laboratories (Riffel et al., 2003; Qiu et al., 2020). Keratin azure is another frequently used substrate in keratinase assay (Lin et al., 1992; Korkmaz et al., 2004; Tork et al., 2016). The enzymatic activity was defined according to the changes of the absorbance at 450 nm after mixing substrate with enzyme for a certain time (Jaouadi et al., 2010; Tork et al., 2016). Keratin azure is commercially available and very suitable for characterization of keratinases with a preference to degrade α-keratins. Keratin-containing materials such as feathers are also frequently utilized in biochemical assays in which feather degradation can be detected through observing the release of amino acids, the shape changes of feathers, and reduction of feather mass (Kim et al., 2005; Anbu et al., 2008; Cãlin et al., 2017; Wu et al., 2017; Bohacz and Korniłłowicz-Kowalska, 2019; Peng et al., 2019). The advantage of using natural keratin is that the keratin-degrading capability of keratinases can be evaluated directly (Gurung et al., 2018; Thankaswamy et al., 2018). The disadvantage is that the substrate degradation requires collaborative effort of different types of enzymes. The native substrate might not reflect the biochemical parameters of a single enzyme. A recent study applied recombinant feather protein in the protease assay. The recombinant protein was soluble in solution, giving rise to accurate analysis of the keratinase (Jin et al., 2017). Therefore, purified enzymes and modified substrates are critical for characterizing the enzymes.

The other type of substrates soluble in solution was applied in the enzymatic assay (Brandelli et al., 2010). Azocasein was utilized in several studies to determine whether a protein harbors protease activity (Tork et al., 2013, 2016). It is a non-specific protease substrate and its hydrolysis results in releases of the azo dye which can be detected by monitoring the absorbance at 366 nm (Tork et al., 2013, 2016). Synthetic peptides were also useful in the protease assays. As the sequence of the peptidic substrates can be modified easily, this type of assay can be utilized to explore the most suitable substrate for a keratinase (Böckle et al., 1995). The cleavage of peptides are able to be monitored using reversed-phase chromatography while this method is not a robust assay (Mäkinen et al., 1994). Peptidic substrates containing p-nitroanilide (pNA) were used to study the preference for P1 and P2 residues (Macedo et al., 2008; Benkiar et al., 2013; Zaraî Jaouadi et al., 2015; Bouacem et al., 2016). As the release of pNA can be detected by measuring the absorbance at 405 nm, pNA derivatives are very suitable to explore the effect of environmental conditions and other chemicals on the activity of keratinases (Rozs et al., 2001). Several keratinases produced by *Bacillus* sp. have been characterized using this type of substrates (Macedo et al., 2008). Fluorogenic substrates containing amino-4-methylcoumarin (AMC) have been widely utilized in the enzymatic assays of serine proteases (Bakhtiar et al., 2005; Silveira et al., 2009). The substrate cleavage can be confirmed with fluorescence spectroscopy. This method is very sensitive and provides an accurate way to characterize proteases. Effect of ions on keratinase activity was explored using AMC-derived substrates (Bakhtiar et al., 2005). Based on accumulated studies, it is a good strategy to adopt multiple protease assays to characterize keratinases obtained from different microorganisms, which will provide an insightful view to these enzymes. The biochemical features of keratinases can be obtained from several reviews (Onifade et al., 1998; Brandelli et al., 2010; Sahni et al., 2015; Kanoksilapatham and Intagun, 2017; Pahua-Ramos et al., 2017; Sharma and Devi, 2018; Zhang et al., 2019; Hassan et al., 2020; Qiu et al., 2020). The crystal structure of rMtaKer provides useful information for the molecular interaction between the enzyme and a peptide sequence from the C-terminal of this enzyme (Figure 4; Wu et al., 2017). This structure will be useful for designing a suitable substrate for enzymatic assays.

In summary, a sensitive biochemical assay needs to be set up as different keratinases may prefer different substrates. In addition, effects of different additives such as reducing reagents on keratin degradation could provide useful insights into understanding keratin degradation and expanding the application of these enzymes.

### Microbial Production of Keratinases

Application of keratinases in industry requires a large amount of enzymes. Therefore, fermentation is essential to produce these enzymes in a large scale to meet the demands from industry (Zaghloul et al., 2011). Fermentation parameters such as carbon source, nitrogen source, temperature, and others need to be optimized as these parameters have an impact on the production of the keratinase. Wastes from industries such as feathers can be added into the cultural medium (Deniz et al., 2021). In addition, other wastes such as wheat and soy beans can be used as a substrate for producing keratinases (Syed et al., 2009; Prakash et al., 2010a). Interestingly, a study showed that glucose and ammonium nitrate are not good sources for growing *Stenotrophomonas maltophilia* to degrade feathers (Qu et al., 2018). Accumulated studies suggest that different microorganisms require various fermentation conditions for large-scale production, indicating that careful exploratory studies are necessary prior to the large-scale production of the enzymes (Jagadeesan et al., 2020; de Menezes et al., 2021; Deniz et al., 2021; Sharma and Kango, 2021). It was demonstrated that the solid-state fermentation increased keratinase production compared with the commonly used submerged fermentation (Inácio et al., 2018). In addition to feathers and human hair used in solid-state fermentation, other low-cost resources from agriculture can be considered in fermentation (Awad et al., 2011).

Recombinant techniques are applied to the production of keratinases (Descamps et al., 2003; Liu et al., 2013b; Fang et al., 2014; Yong et al., 2020; Yahaya et al., 2021). This method is particularly meaningful for keratinases that are produced by pathogenic microorganisms (Muhammed et al., 2021) and the mutants with an improved enzymatic activity and stability (Zhang et al., 2020). The recombinant production of keratinases does not require the application of keratin as the carbon and nitrogen sources. It is possible to purify the recombinant enzymes in a fast way when an affinity purification tag is introduced. Several studies demonstrate that it is feasible to produce recombinant keratinases. Keratinases from bacteria can be produced in *Escherichia coli* (Tiwary and Gupta, 2010a). It has been shown that the gene kerA encoding a keratinase from *Bacillus licheniformis* was expressed in *Escherichia coli* and *Bacillus subtilis* while the yield was lower than that of the wild type. An improved yield was observed by integration of multiple copies of kerA into the chromosome (Wang et al., 2004). Therefore, producing keratinases using recombinant techniques is of great interest while extensive studies are still needed to obtain the recombinant keratinase with an improved activity.

The purification of keratinases is important for enzymatic characterization and other application (Brandelli et al., 2015). In waste treatment, the enzyme purification is not needed for reducing cost and improving efficiency. To obtain a keratinase with a high purity, several strategies can be utilized. Ammonium sulfate precipitation, gel filtration chromatography, and ion-exchange chromatography are commonly used methods in the purification (Brandelli et al., 2015). For recombinant proteins, the affinity chromatography can be used in purification based on the affinity tag that is incorporated into the target protein. Keratinases from different bacteria have been purified for biochemical characterization. Techniques such as the aqueous two-phase system are applicable to obtain a large amount of enzymes (Bach et al., 2012; Sala et al., 2014). A carefully experimental design is vital when a large quantity of pure enzymes is needed as the purification could be an expensive step.

It has been noted that recombinant techniques are still needed for producing keratinases with a high purity, keratinases with mutations, and keratinases originated from a pathogenic microorganism (Liu et al., 2014). Recombinant protein expression systems and host and gene cloning strategies need to be explored (Gong et al., 2020). As the recombinant protein is critical for exploring the function of keratinases, it is useful in studying the function and activity of the enzymes.

## Application of Keratinases

**FIGURE 5:**
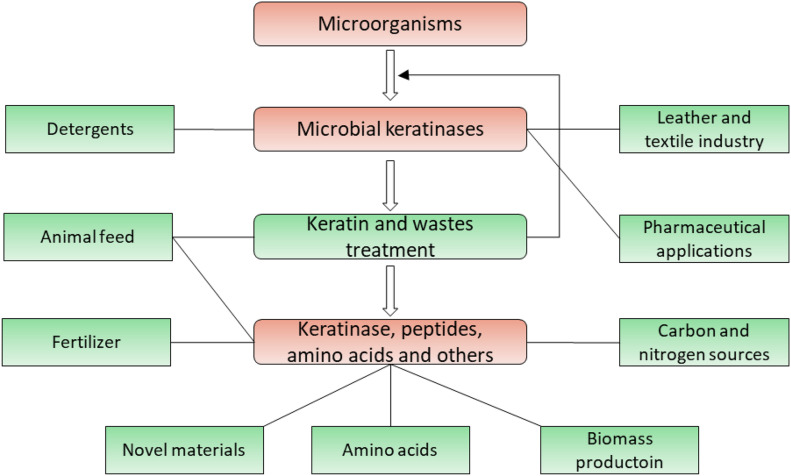
Application of microbial keratinases in different fields. Applications of keratinases and their products are highlighted in green, which has been introduced in multiple reports (Onifade et al., 1998; Gupta and Ramnani, 2006; Brandelli et al., 2010; Chaturvedi et al., 2014; Sahni et al., 2015; Sharma and Gupta, 2016; Vidmar and Vodovnik, 2018).

Keratin exists widely in nature and is a valuable source of carbon, nitrogen, and sulfur which can be converted into diverse products (McKittrick et al., 2012; Wang et al., 2016). Keratinases have extensive industrial and biotechnological applications due to their ability to degrade keratins (Onifade et al., 1998; Gupta and Ramnani, 2006; Rai et al., 2009; Syed et al., 2009; Brandelli et al., 2010; Sahni et al., 2015; Kanoksilapatham and Intagun, 2017; Gegeckas et al., 2018; Vidmar and Vodovnik, 2018). Their applications are summarized (Figure 5) and have been described in several literatures (Gupta and Ramnani, 2006; Brandelli et al., 2010; Vidmar and Vodovnik, 2018; Hassan et al., 2020; Nnolim and Nwodo, 2021).

## Improvement of Keratinases

To improve the activity and thermal stability of keratinases, mutagenesis was applied (Fang et al., 2010; Wang et al., 2015; de Paiva et al., 2018; Su et al., 2019). The mutagenesis studies were carried out by treating whole cells with reagents and modifying genes using molecular biology. In a study, ethyl methanesulfonate (EMS) was used to treat the whole cells of *Bacillus subtilis* LFB-FIOCRUZ 1266, and the resulting mutant strains exhibited higher feather hydrolysis compared with the wild-type strain (de Paiva et al., 2018). Treatment of feather-degrading *Deinococcus ficus* with UV resulted in mutants with an improved and decreased keratinolytic activity (Zeng et al., 2011). Other chemical reagents such as ethidium bromide (EtBr) and *N*-methy-*N*’-nitro-*N*-nitroso-guanidine (MNG) were also able to cause mutations in the genes, which affected the keratinase activity (Cai et al., 2008; Vidmar and Vodovnik, 2018).

Protein engineering was also applied to cause an augmentation of the keratinase activity (Fang et al., 2019). When the amino acid sequence and structure of a keratinase are available, the rational protein design can play a role in improving the activity and thermal stability of a keratinase. When amino acids essential for the protease activity, metal binding, and thermal stability are identified, computer-based methods will enable researchers to design proteins with improved enzymatic activities and thermal stabilities. This strategy has been successfully applied to the keratinase of *Bacillus licheniformis* BBE11 (Liu et al., 2013a). Four amino acid substitutions (N122Y, N217S, A193P, N160C) were designed, and the corresponding genes were expressed in *Bacillus subtilis* WB60. A mutant keratinase with the N122Y substitution exhibited an approximately 5.6-fold increase in catalytic efficiency, suggesting that this is an efficient strategy in improving activity and stability (Liu et al., 2013a). Other methods such as PCR-based methods and direct evolution will play a role in obtaining more potent keratinases (Vidmar and Vodovnik, 2018). When the regulatory mechanism of a keratinase is understood, the modification on other regions of the keratinase can also improve its activity and stability (Fang et al., 2016b; Peng et al., 2021). In a study, the N- and C-terminal regions of KerSMD were replaced with those regions of a homogenous keratinase. Replacing the N-terminal region resulted in a mutant exhibiting more than a twofold catalytic activity toward casein catalytic efficiency. Replacing the C-terminal region improved keratinases activity using succinyl-Ala-Ala-Pro-Phe-p-nitroanilide as a substrate in a biochemical assay. Replacing both N- and C-terminal regions resulted in a mutant with an improved thermal stability (Fang et al., 2016b).

It is important to improve the enzymatic characteristics of the keratinase while caution has to be taken when the whole-cell-based mutagenesis is used. All the mutant strains should meet the safety requirement from certain authorities. Compared with random mutagenesis, structure-guided protein engineering is of great interest as the mutation is well managed. To carry out such studies efficiently, the amino acid sequence and structures need to be known. Recombinant protein production is therefore a strategy to play an important role in this process. Researchers have to make sure that the strains with modified genes are acceptable in industrial applications.

## Conclusion

Keratin is a rich resource in nature, and the amount of keratin-rich wastes is increasing annually. Keratinases play important roles in keratin recycle and have diverse applications in different fields. Studies need to be carried out to obtain active enzymes and enlarge their applications. Microbiology, molecular biology, structural biology, computation and biochemistry will play important roles in the research field of keratinases.

## Author Contributions

QL drafted and revised the manuscript.

## Conflict of Interest

The author declares that the research was conducted in the absence of any commercial or financial relationships that could be construed as a potential conflict of interest.

## Footnotes

1 https://patents.google.com/
